# Sedentary Behavior: A Key Component in the Interaction between an Integrated Lifestyle Approach and Cardiac Autonomic Function in Active Young Men

**DOI:** 10.3390/ijerph16122156

**Published:** 2019-06-18

**Authors:** Renan R. dos Santos, Erica C. Rosa, Thiago Rosa, Eduardo A. Ferreira, Eliana F. Gris, Rosângela V. de Andrade, Angélica A. Amato

**Affiliations:** 1 Postgraduate Program in Health Sciences, University of Brasília, Brasília 70904-970, Brazil; erica.cccaldas@gmail.com (E.C.R.); angelicamato@unb.br (A.A.A.); 2 Postgraduate Program in Physical Education, Catholic University of Brasilia, Brasília 71966-700, Brazil; thiagoacsdkp@yahoo.com.br; 3 Faculty of Pharmacy of Ceilândia, University of Brasília, Brasília 72220-275, Brazil; eduardoantonioferreira@gmail.com (E.A.F.); elianagris@gmail.com (E.F.G.); 4 Postgraduate Program in Genomic Sciences and Biotechnology, Catholic University of Brasilia, Brasília 70790-160, Brazil; rosangelavand@gmail.com

**Keywords:** physical activity, sedentary behavior, sleep quality, heart rate variability

## Abstract

This study aimed to verify the association between autonomic cardiac function (CAF) and the integration of caloric expenditure by physical activity (PA) intensity, sedentary behavior (SB), and sleep quality (PSQI) in active young men. Thirty-five subjects were included, and caloric expenditure in moderate-to-vigorous and light-intensity PA, SB, and PSQI were assessed using questionnaires. Heart rate variability (HRV) was recorded for short periods of time in the supine and orthostatic positions. Multiple linear regression was realized unadjusted and adjusted for covariables, such as age, body mass index, and fat mass. No adjusted analysis indicated that, in the supine position, there were negative associations between the SB and the TP, HF, and NorHF indices, and positive associations between SB and NorLF and LF/HF. In the orthostatic position, an interaction between SB and NorLF was found. Significance of proportion with the TP, HF, and LF/HF indices was confirmed. When adjusted, for the supine position, negative interactions were documented between SB and the TP as well as the HF indices, and between PSQI and the LF/HF index, with interference under the HF and LF/HF indices. Finally, our findings indicate that the proposed approach interacts with CAF, and SB is significantly related to CAF in young active men.

## 1. Introduction

A healthy lifestyle is a key component to promote healthy aging and to reduce the risk of cardiovascular events, entailing, among other aspects, the engagement in regular physical activity (PA), reduced sedentary behavior (SB), and good sleep quality [1,2,3].

The autonomic nervous system (ANS) has an important role in homeostasis, modulating blood pressure, heart rate, and peripheral vascular resistance. Moreover, ANS function is an important indicator of health, both at rest and in response to different stimuli [4]. Heart rate variability (HRV) is a non-invasive and low-cost method for assessment of cardiac neuromodulation [5] and can predict a higher risk of cardiovascular morbidity and mortality [6,7].

Healthy adults with an active lifestyle demonstrate higher HRV than their sedentary peers [8]. However, high levels of SB may counteract the favorable effects of an active lifestyle, by promoting harmful HRV levels at rest, suggesting an autonomic imbalance [9,10]. ANS also plays a key role in sleep physiology, and there is fluctuation between sympathetic and parasympathetic dominance during transition between the different sleep stages [11]. This indicates that attenuated HRV is an important predictor of poor sleep quality in subjects with chronic fatigue syndrome [12]. Indeed, Hall et al. [13] point out that acute psychophysiological stress affects HRV during sleep in healthy adults, promoting higher sympathetic predominance and poor sleep maintenance. This can lead to a myriad of disorders and a decreased quality of life.

Taken together, these findings indicate that SB and poor sleep quality have deleterious effects on health, regardless of the level of PA [14,15] and that this effect may involve cardiac autonomic function (CAF). In addition, some health authorities in Australia, the United Kingdom and, the United States provided recommendations to reduce daily sitting time among children and adults [16,17,18]. However, little is known about the impact of SB on CAF in a scenario combined with lifestyle behaviors. Thus, this study aimed to investigate the association between CAF and an integrated approach to lifestyle aspects, such as caloric expenditure by PA intensity, SB, and sleep quality in active young men. Our growing understanding of the relationship between these aspects and cardiovascular physiology may provide substantial information from which future interventions may be aimed at positive changes in PA, SB, and sleep.

## 2. Materials and Methods

### 2.1. Sample

The sample consisted of 35 young adults who satisfied the following criteria: 18–40 years old; male; body mass index (BMI) between 18.5 and 30 kg/m^2^; nonsmokers considered to be low health risk according to medical history [19]; free of apparent diseases (such as heart disease, hypertension, diabetes, or other chronic diseases) or physical disorders (as determined by a subjective questionnaire); physical activity levels of ≥150 min/week in the last six months (IPAQ); and an absence treatment with drugs that could affect the outcomes assessed (adrenergic, anti-adrenergic, cholinergic, anti-cholinergic).

In addition, statistical power calculations that were previously performed indicating that a sample of 35 participants is sufficient for a model with five predictors to detect association values with an effect size (*r*^2^) of up to 0.25 (average) for an adequate statistical power of 0.80 and a level of significance of 0.05.

This study was approved by the Committee of Ethics in Research with Human Beings of the Faculty of Health Sciences of the University of Brasilia, according to CEP-FS No. 1.660.676, based on Resolution 196/96 of the National Health Council of the Ministry of Health, which regulates human research in Brazil, and all participants gave written informed consent [20].

### 2.2. Experimental Design

The participants made one visit to the laboratory where the evaluation occurred in a quiet environment, from 02:00–04:00 pm, with temperatures set between 22 °C and 25 °C. All participants were instructed to refrain from stimulants, alcoholic drinks, medication, and physical activity for at least 24 h prior to the study.

At first, participants answered questionnaires about their PA and SB levels, PSQI, and anamnesis for risk stratification. Then, anthropometric data (weight, height, percentage of fat mass) and basic physiological data were collected. The volunteers laid on a stretcher for a period of 10 minutes, after which a three-minute electrocardiogram was recorded at rest and the presence of sinus node rhythm was verified, which, when found, started to record the HRV. The evaluation occurred in the baseline condition, while the volunteers were at rest in the supine and orthostatic position. Once they had maintained this position for 10 min, their HRVs were recorded for a period of five minutes. Subsequently, participants were asked to adopt the orthostatic posture at the edge of the bed; two minutes after assuming this position, volunteers’ HRVs were recorded for an additional five minutes [21,22].

### 2.3. Instruments

#### 2.3.1. Physical Assessment

Measurements of weight (kg) and height (cm) were performed with Filizola-Model 31 scale; percentage of fat mass (%FM) by Bio-eletrical Impedance (Omron BF306, Omron Healthcare Co., Ltd., Kyoto, Japan); and BMI (kg/m^2^). Polar© (V800, Polar Electro Oy, Finland) was used to the characterization of heart rate (HR) and Microlife© (Microlife Corp., Taipei, Taiwan) was used to gauge systolic and diastolic blood pressure (SBP and DBP, respectively). Mean arterial pressure (MAP) was calculated by the formula DBP + (SBP − DBP)/3 [23]. The volunteers were evaluated in two positions: supine position (basal) and orthostatic position (both of which were used for the autonomic cardiac function test). In each situation, the values measured after the first minute were considered.

#### 2.3.2. Physical Activity and Sedentary Behavior

The International Physical Activity Questionnaire (IPAQ), short form, version 8, was used to quantify the level of physical activity. IPAQ is recommended for national prevalence studies because it makes international comparison possible. This version contains eight items related to the frequency (days per week) and duration (times per day) of vigorous, moderate and light intensity physical activity, as well as the frequency and duration of sedentary behavior (such as sitting) during the week [24,25].

To calculate the weekly energy expenditure of each study volunteer, their energy expenditure was multiplied according to the activity performed, considering the weekly frequency and the duration of the energy expenditure (average time in minutes/week).

To convert the data obtained through IPAQ into metabolic equivalent (MET), the average for each intensity domain proposed by Heymsfield [26] was used, with the following values taken into consideration for each domain: light = 3.3 METs, moderate = 4.0 METs, and vigorous = 7.1 METs.

By multiplying the MET value of the activity performed by the weekly frequency and duration, the caloric expenditure in MET minutes/weeks was calculated. To convert to kilocalories (kcal), the value obtained was multiplied by the weight and divided by 60 min. Thus, the caloric expenditure value of each activity was expressed in kcal during the week [26].

#### 2.3.3. Sleep Quality

Sleep quality was assessed using the Pittsburgh Sleep Quality Index (PSQI). Each score is assigned a certain number of points; the maximum score is 21 points. Scores higher than 5 indicate poor sleep quality [27]. Once this instrument was developed and validated [28], it presented a sensitivity of 89.6% and a specificity of 86.5%. When translated and validated in Portuguese, it maintained its high sensitivity of 80% while demonstrating a specificity slightly lower than 68.8% [29].

#### 2.3.4. Heart Rate Variability Analysis

The automatic generation of the time series of the R-R intervals was performed using the POLAR^®^ V800 [30]. The time series records for HRV analysis at rest were collected and transformed into a text file (.txt) by online software Polar Flow. The .txt (data) files were processed and the autonomic cardiac response analyzed by means of a specific software function (Kubios HRV©, (Kubios HRV v2.0, University of Kuopio, Finland)).

Prior to HRV analysis, each series of R-R intervals was visually checked on a beat-to-beat basis for sinus rhythm validation and identification of non-sinusoidal and ectopic beats, artifacts, and signal reliability. Spurious, occasional, and possibly discrepant beats were excluded from the series without adding new ranges. The R-R range series was highly stable and stationary, as estimated by the percentage differences of means and standard deviations between three divided segments of the series.

The HRV was analyzed in the frequency domain by means of different established standard indices [5,6]. The following indices were obtained: the total power spectral area (TP) comprising the entire spectrum of frequencies, up to the maximum limit of 0.50 Hz, which expresses the global autonomic modulation; low (LF: 0.04–0.15) and high (HF: 0.15–0.50 Hz) spectral frequencies, which express, respectively, the combined sympathetic and vagal modulation as well as the predominantly parasympathetic modulation; the ratio between the absolute areas of the bands of low and high spectral frequencies (LF/HF) that estimate the sympatho-vagal balance, and the normalized power areas of low (NorLF) and high (NorHF) frequency bands, which were the percentage of the absolute power area of each band in relation to the sum of both absolute areas. Within this analysis, individuals can be classified in terms of the balance between the sympathetic and parasympathetic portions of the ANS, according to the value obtained in the ratio between the absolute areas of low and high frequency bands, namely: LF/HF ratio > 1: “sympathicotonic” (sympathetic predominance); LF/HF ratio = 1: “anphotonic” (sympatho-vagal balance); LF/HF ratio < 1: “vagotonic” (parasympathetic predominance). All analyses followed the recommendations of the task force instituted in 1996 for HRV studies [5].

### 2.4. Statistical Analysis

Data were expressed as median and interquartile ranges (25–75%). The comparison between the different positions (supine vs. orthostatic) and their descriptive variables was made based on the crude values, using the Wilcoxon paired test.

For multiple linear regression, several procedures were adopted. Initially, the data had normality and homogeneity verified by Shapiro-Wilk and Levenne tests, respectively. Since normality and homogeneity were not verified for most variables, all data were transformed into log10 and retested. With this, the normality and homogeneity of the data were confirmed. The multicollinearity between the independent variables was verified through the Pearson correlation coefficient and the multicollinearity index to establish the best model.

All variables inserted in the models were selected based on primary theoretical links and subsequently adjusted based on the fact that they did not violate statistical laws [31]. Based on these parameters, multiple linear regression analysis was performed between the crude model that was above described which took into consideration (i) caloric expenditure per week, reported in moderate-to-vigorous PA, and caloric expenditure per week, reported in light intensity PA; (ii) SB; and (iii) PSQI with HRV indices, and then adjusted for a hierarchical regression analysis with the introduction of the following covariates: age, BMI, and FM percentage (Block 1) and caloric expenditure per week, reported in moderate-to-vigorous PA and caloric expenditure per week reported in light intensity PA, SB, and PSQI (Block 2) for each HRV index, the standardized regression coefficient (Beta) and its significance (p) were calculated in relation to the crude model and the adjusted model. For the crude model, the size of the effect according to *r*, *r*^2^, *F*, and the significance value of *F* were attested to verify the pertinence of the crude model for each studied variable. For Blocks 1 and 2, the coefficient of determination *r*^2^ was used, as well as its percentage, in order to determine the proportion of variation explained by each block of variables added.

The significance level adopted was *p* < 0.05 and *F* < 0.05.All procedures were performed using the Statistical Package for Social Sciences 22.0^®^ (SPSS 22.0, Chicago, ILL, USA) and G * Power 3.0^®^ (Heinrich-Heine-Universität Düsseldorf, Düsseldorf, Germany) programs.

## 3. Results

Participants were considered active (>150 min/week) and had higher caloric expenditure in vigorous and moderate intensity activities than in the light intensity domain. These participants were also considered to have low levels of SB (<3360 min/week) and were classified with good sleep quality (Table 1).

Table 2 presents the hemodynamic parameters (HR, SBP, DBP, and MAP) verified in the two different positions (supine and orthostatic). There was a significant increase in HR and SBP after standing (*p* < 0.05). About HRV indices, after standing there was a reduction of predominantly vagal control (HF and NorHF), and an increase in sympathetic predominance (LF and NorLF). Moreover, in the supine position the participants showed an “anphotonic” characteristic (LF/HF = 1.12) whereas, in the orthostatic position, a “sympathicotonic” condition was observed (LF/HF = 6.16).

When analyzing the crude model in relation to HRV (Table 3), in the supine position, there was a significant association between SB and several HRV indices. This interaction was negative for the indices TP (Beta = −0.44, *p* = 0.00), HF (Beta = −0.63, *p* = 0.00) and NorHF (Beta = −0.38; *p* = 0.01), and positive for the indices: NorLF (Beta = 0.31, *p* = 0.04) and LF/HF ratio (Beta = 0.53, *p* = 0.00). after assuming the orthostatic position, there was a positive and significant association with the indication “sympatho-vagal” NorLF index (Beta = 0.3, *p* = 0.04).

**Figure 1:**
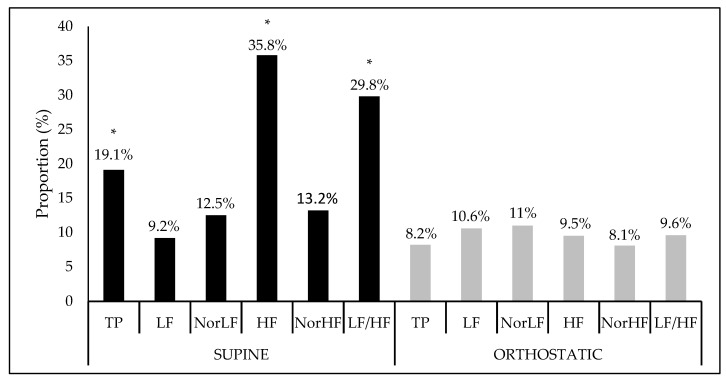
Interference proportion (%) of the crude model apllied at the heart variability índices in the supine and orthostatic position. TP: Total power area; LF: Low frequency; NorLF: Normalized low frequency; HF: High frequency; NorHF: Normalized high frequency; LF/HF: Ratio low frequency/high frequency. * Significance at *F* < 0.05.

Figure 1 shows the interference proportion and the significance of the crude model for each HRV index. In a supine position, there was a statistical significance of the model for the indices: TP (*F* = 2.65, Sig. *F* = 0.04), HF (*F* = 6.28, Sig. *F* = 0.00), and LF/HF ratio (*F* = 4.77, Sig. *F* = 0.00) while, in the orthostatic position, no significant interactions were found.

Regarding the analysis of the adjusted model (Table 4), in the supine position, we observed that SB was negatively associated with the TP (Beta = −0.58, *p* = 0.03) and HF (Beta = 0.50, *p* = 0.03), and with the sleep quality, which presents significant and negative scores with the LF/HF ratio (Beta = −0.30, *p* = 0.02). In the orthostatic position, no significant associations were found.

**Figure 2:**
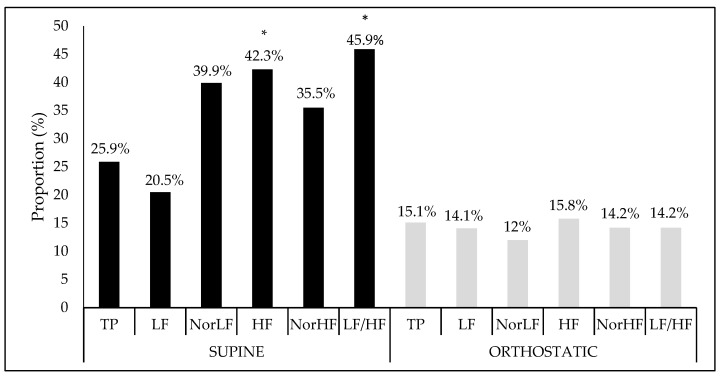
Interference proportion (%) of crude model adjusted to covariables in relation at the heart rate variability indices in the supine and orthostatic position. TP: Total power area; LF: Low frequency; NorLF: Normalized low frequency; HF: High frequency; NorHF: Normalized high frequency; LF/HF: Ratio low frequency/high frequency.* Significance at *F* < 0.05.

Finally, when the proportion of interference and the crude model’s significance were verified together with their covariates (age, BMI, and fat percentage), as Figure 2 shows, in the supine position, there was significant interference between the analysis performed and both the vagal HF component (*F* = 2.97, Sig. *F* = 0.03), and the sympatho-vagal balance LF/HF ratio (*F* = 2.66, Sig. *F* = 0.04). However, no significant association was found in the orthostatic position.

## 4. Discussion

The present study verified whether a lifestyle approach combining caloric expenditure in vigorous and moderate intensity PA, with caloric expenditure in low intensity PA, SB and sleep quality are associated with autonomic cardiac function. However, some aspects should be emphasized: (1) the change of body position generated significant stress on the hemodynamic parameters evaluated, confirming differences in organic control in the positions studied; (2) SB showed greater importance than the other analyzed aspects, interacting in a damaging way the ANS activity, favoring greater sympathetic activity, and decreasing parasympathetic activity in the supine and orthostatic rest positions. As even with the addition of covariates, this aspect still remains significant; (3) sleep quality presented a negative interaction with the sympatho-vagal balance in the supine rest position and; (4) the lifestyle approach used in this study showed a significant interaction with the CAF, both in an isolated manner and in a covariate set, as the main mediator under the vagal component.

### 4.1. Hemodynamic Variables and Heart Rate Variability in the Different Positions

Changes in body position lead to adjustments to maintain homeostasis [32]. However, the mechanism by which this process occurs is varied and depends, among other factors, on the individual’s age and physical condition. As indicated by Laitinen et al. [33], in young subjects, HR increased without a concomitant increase in MAP and cardiac output as a mode of organic adjustment in function of postural change. In the same study, however, no changes were observed in HRV parameters in healthy men and women. On the other hand, Carnethon et al. [34] observed changes in hemodynamic parameters (HR and BP) in elderly subjects who exhibited risk factors for cardiovascular disease.

Nevertheless, the findings of the present study agree with the subject matter literature as a general physiological response for healthy adults, as indicated by the studies of Acharya et al. [35], Gilder and Ramsbottom [36] and Chan et al., [37], which observed in populations with these characteristics that when submitted a change of position (supine for orthostatic), higher HR, and lower HRV as adjustments of the cardiovascular system to the imposed challenge.

### 4.2. Unadjusted Model and Heart Rate Variability

The association of each variable of the crude model with the HRV indices show/highlight that SB was the variable with the most prominent influence on HRV of active young men, anticipating a reduction of vagal modulation in the supine position for a more “sympathicotonic” profile at rest and a greater sympathetic component (NorLF) in the orthostatic position due to this habit. In this sense, the literature has enriched our understanding of this theme over the course of the previous century, and it is a well-known fact that independent of the level of PA the excessive exposure to SB is associated with an increased risk of mortality [14,38,39]. According to Stamatakis et al. [40], the association of SB with long-term outcomes may contribute to guidelines for reducing the amount of time spent during the day on this type of behavior. As indicated by Kartzmarzyk et al. [39] in a meta-analysis study, there is a two-year increase in life expectancy when time spent in a seated position is reduced to less than 3 h/day; there is an increase of 1.38 years when sedentary behavior is limited to less than 2 h/day of TV viewing.

However, studies analyzing the mechanisms underlying the deleterious effects of SB are still lacking, and SB possibly acts independently from PA to affect health. Recent studies have shown that when SB is interspersed with sudden bursts of PA, it may be an effective alternative to improve cardiovascular outcomes [41,42]. Although this was not the focus of the present study, the fact is the participants presented characteristics that follow the recommendations, which may be the way by which interventions should be directed preventively in healthy populations.

Chau et al. [38] conducted a meta-analysis of 595,086 participants in six previous studies in which they examined the association between total time seated and all-cause mortality. They found that despite the deleterious effects of SB, PA appears to act in a partially protective manner, especially in individuals who spend a lot of time sitting during the day. Rees-Punia et al. [43], however, indicate that when highly active subjects are considered the SB replacement for longer time from a moderate-to-vigorous intensity PA, it does not appear to provide additional benefits for all-cause mortality.

The relative lack of significance of the results of the present study as for the case of the variables adopted for caloric expenditure by intensity of PA, which in turn was determined by the PA levels (vigorous, moderate, light) of the sample investigated [15]. Future studies with the same sampling characteristics should be undertaken to verify how SB acts when used as the primary intervention. In this context, Hallman et al. [9] directly linked SB to autonomic cardiac function and showed a negative association between HRV and occupational (rather than leisure time) SB among blue-collar workers. While their results corroborate those of the present study, they emphasize that SB has nuances that result in differentiated physiological behaviors that require further exploration.

It must be emphasized that sleep interference in the ANS and sleep quantity have a paradoxical relationship—extended sleep time is positively associated with SB, while short sleep time is associated with poor quality of life and diverse cardiovascular outcomes [44]. Roach et al. [45], verified the efficacy of divided work regimens by comparing the quantity/quality of sleep obtained by participants who adhered to a divided sleep-wake protocol with that of those who adhered to a consolidated protocol. Their study did not reveal any differences between the two protocols; instead, it showed that the division of sleep into stages during the day is an adequate alternative for the “restoration” of the organism. In contrast, Van Dongen et al. [46] demonstrated that accumulated sleep loss has deleterious effects on the neurobehavioral functions of wakefulness in healthy adults. Basset et al. [47] concluded that sleep quality, not quantity, affects the response of the hormone cortisol as a function of acute psychosocial stress in young adults. Already, Simpson et al. [48] emphasize that a better quality of sleep positively affects several outcomes related to the physical and cognitive performance of athletes. Sleep is a complex physiological condition; the variations in sleep stages and the autonomic control associated with behavioral habits require further study.

The integrated lifestyle approach used in this study confirms the significance of the interaction between global cardiac autonomic modulation and the vagal component. Although the lifestyle components have already been addressed in other studies [49,50,51], the analysis proposed here is innovative and relevant for future studies related with cardiovascular physiology and lifestyle interventions.

### 4.3. Adjusted Model and Heart Rate Variability

The adjustment of the lifestyle approach for age and body composition parameters (Percentage of fat and BMI) pointed out in this study presents negative interactions of SB with the global modulation TP index and with the predominance of the vagal HF index, the interaction between the sleep quality and the “sympathovagal” LF/HF ratio was similar. In this sense, current literature presents evidence that age is inversely related to autonomic control [52,53,54]. Regarding body composition parameters, Arora et al. [55] examined the direct and indirect impact of sleep quantity and quality on insulin resistance, with BMI as a potential mediator in patients with type 2 diabetes mellitus. They concluded that sleep quality plays an important role in insulin resistance and that BMI mediates this relationship. Rahe et al. [56], when investigating the association between sleep quality and different markers of obesity (general obesity, abdominal obesity, and fat percentage) in 753 adult subjects, revealed that this factor can predict obesity, as well as the high percentage of fat mass.

Kahlhöfer et al. [57], examined the association between objectively measured sleep (sleep quality and quantity) and body composition in 127 young adults (similar to the present study). However, their analysis was conducted considering PA, eating habits, and autonomic function as potential determinants of this relationship. Revealing that, beyond sleep quality being inversely associated with fat percentage, SNA can be a mediator of this process, because the LF/HF ratio and the sympathetic indicator LF were positively related to the high percentage of fat mass in this population. Therefore, when covariates of an important outcome for morbidity and mortality are considered, the association between sleep quality and the LF/HF ratio has gained impetus and is important to be taken into account in future studies in relation to the behavior of the ANS as a mediator of behavioral processes that are deleterious when poorly structured in a routine life.

It is also believed that the increase in relative values and the maintenance of significance when the ratio of interaction is verified between the adjusted model and HRV, more specifically with the vagal HF indices and the LF/HF ratio, confirm the importance of the aforementioned associations in a global context of interaction between the cardiovascular system and physiological behavior.

### 4.4. Limitations of the Study

This study has several methodological limitations. First, all lifestyle aspects’ data were extracted from self-reported questionnaires. Alternative tools with the same validity and reproducibility have not yet been discovered. Second, the statistical transformation of the data into Log10 does not allow the creation of formulas that estimate the HRV values from the behavioral data, so that the estimation of the data would only serve the values in Log10 and that, later, when reconverted to the data, already lose their association. In this case, the transformation had the appropriate statistical support and was based on answering the question proposed here; however, estimating “non-normal” data from codes is a step no longer possible with the results obtained here. Finally, the limited sample size makes it difficult to generalize the results.

## 5. Conclusions

This study verified that an integrated lifestyle approach by caloric expenditure in physical activities of vigorous and moderate intensities, caloric expenditure in light intensity physical activities, sedentary behavior, and sleep quality are associate with cardiac autonomic function, assessed by heart rate variability indices in young active men. In this population, sedentary behavior was the main lifestyle-related factor associated with autonomic cardiac function at rest.

## Figures and Tables

**Table 1:** Descriptive information of the study participants (*n* = 35) regarding the behavioral parameters addressed.

Variable	Median (IQ 25%–75%)
PSQI	5 (4–6)
Physical Activity Level (min/wk)	440 (300–640)
Caloric expenditure in vigorous and moderate intensity physical activity (kcal/wk)	2356 (1623–3822) ^†^
Caloric expenditure in light intensity physical activity (kcal/wk)	120 (0–450) ^†^
Sedentary behavior (min/wk)	2580 (1800–4260)

PSQI: Sleep quality index. ^†^ *p* < 0.05 when compared to caloric expenditure in vigorous and moderate physical activity vs. low intensity.

**Table 2:** Median and interquartile range (25–75%) of the hemodynamic parameters and the Heart Rate Variability in the supine and orthostatic position.

Variable	Supine	Orthostatic	*p*
HR (bpm)	58.11 (54.32–63.92)	71.93 (67.53–75.20)	<0.001
SBP (mmHg)	122.0 (115.0–134.0)	117.0 (107.0–133.0)	0.013
DBP (mmHg)	70.0 (64.0–76.0)	69.0 (64.0–77.0)	0.825
MAP (mmHg)	89.33 (80.33–94.0)	85.33 (78.0–94.0)	0.261
TP (ms^2^)	4120 (2535–7412)	3943 (2493–6093)	0.883
LF (ms^2^)	1339 (792–2288)	2170 (1122–3608) *	0.005
NorLF (%)	52 (40.8–67.5)	86 (77.8–91.9) *	<0.001
HF (ms^2^)	1237 (448–2184)	328 (193–715) *	<0.001
NorHF (%)	47.9 (32.2–59.1)	14 (8.1–22.1) *	<0.001
LF/HF	1.12 (0.76–2.37)	6.16 (3.52–11.38) *	<0.001

HR: Heart rate; SBP: Systolic blood pressure; DBP: Diastolic blood pressure; MAP: Mean arterial pressure; TP: Total power area; LF: Low frequency; NorLF: Normalized low frequency; HF: High frequency; NorHF: Normalized high frequency; LF/HF: Ratio low frequency/high frequency. * Significant difference between the median value in the supine compared to the orthostatic position at *p* < 0.05.

**Table 3:** Standardized regression coefficient (Beta) and significance (*p*) for each analyzed variable of the crude model, in relation to the heart rate variability in the supine and orthostatic positions.

Variable	EKcal Vig-Mod (Kcal/Wk)		EKcal Light (Kcal/Wk)		Sedentary Behavior (Min/Wk)		Sleep Quality (PSQI)	
	Beta	*p*	Beta	*p*	Beta	*p*	Beta	*p*
SUPINE								
TP (ms^2^)	0.08	0.54	−0.16	0.25	−0.44	0.00 *	0.19	0.20
LF (ms^2^)	0.01	0.97	−0.19	0.20	−0.28	0.07	0.07	0.64
NorLF (%)	0.09	0.55	−0.13	0.37	0.31	0.04 *	−0.12	0.42
HF (ms^2^)	−0.07	0.59	−0.09	0.46	−0.63	0.00 *	0.22	0.09
NorHF (%)	−0.05	0.71	−0.02	0.91	−0.38	0.01 *	0.09	0.54
LF/HF	0.10	0.45	−0.10	0.44	0.53	0.00 *	−0.21	0.12
ORTHOSTATIC								
TP (ms^2^)	0.17	0.24	−0.12	0.40	−0.21	0.17	0.10	0.50
LF (ms^2^)	0.22	0.13	−0.05	0.74	−0.26	0.09	0.13	0.39
NorLF (%)	0.09	0.55	−0.13	0.37	0.31	0.04 *	−0.12	0.42
HF (ms^2^)	−0.02	0.89	−0.18	0.23	−0.28	0.07	0.02	0.89
NorHF (%)	−0.23	0.13	−0.14	0.34	−0.10	0.52	−0.07	0.66
LF/HF	0.23	0.11	0.14	0.33	0.15	0.33	0.05	0.73

EKcal: Caloric expenditure; TP: Total power area; LF: Low frequency; NorLF: Normalized low frequency; HF: High frequency; NorHF: Normalized high frequency; LF/HF: Ratio low frequency/high frequency. * Significant interaction between the HRV index and the behavior score for *p* < 0.05.

**Table 4:** Standardized regression coefficient (Beta) and significance (*p*) values for each variable of crude model adjusted with covariates (Age, BMI and Percentage fat mass) in relation to the heart rate variability indices in the supine and orthostatic positions.

Variables	EKcal Vig-Mod (Kcal/Wk)		EKcal Light (Kcal/Wk)		Sedentary Behavior (Min/Wk)		Sleep Quality (PSQI)	
	Beta	*p*	Beta	*p*	Beta	*p*	Beta	*p*
SUPINE								
TP (ms^2^)	0.15	0.30	−0.17	0.22	−0.58	0.03 *	0.14	0.33
LF (ms^2^)	0.09	0.55	−0.17	0.25	−0.50	0.06	0.01	0.96
NorLF (%)	0.12	0.33	−0.01	0.97	−0.24	0.29	−0.22	0.11
HF (ms^2^)	−0.03	0.78	−0.14	0.27	−0.50	0.03 *	0.22	0.09
NorHF (%)	−0.10	0.45	−0.12	0.35	0.12	0.62	0.18	0.19
LF/HF	0.15	0.22	−0.01	0.92	0.09	0.69	−0.30	0.02 *
ORTHOSTATIC								
TP (ms^2^)	0.23	0.13	−0.08	0.60	−0.56	0.43	0.03	0.84
LF (ms^2^)	0.24	0.11	−0.01	0.97	−0.52	0.06	0.08	0.61
NorLF (%)	0.19	0.22	0.17	0.26	0.07	0.79	0.22	0.17
HF (ms^2^)	0.02	0.88	−0.15	0.33	−0.41	0.13	−0.02	0.92
NorHF (%)	−0.19	0.19	−0.14	0.34	−0.01	0.97	−0.07	0.66
LF/HF	0.21	0.16	0.13	0.38	0.11	0.69	0.06	0.72

EKcal: Caloric expenditure; TP: Total power area; LF: Low frequency; NorLF:Normalized low frequency; HF: High frequency; NorHF: Normalized high frequency; LF/HF: Ratio low frequency/high frequency. * Significant interaction between the HRV indice and the behavior score for *p* < 0.05.
